# Rapid energy expenditure estimation for ankle assisted and inclined loaded walking

**DOI:** 10.1186/s12984-019-0535-7

**Published:** 2019-06-06

**Authors:** Patrick Slade, Rachel Troutman, Mykel J. Kochenderfer, Steven H. Collins, Scott L. Delp

**Affiliations:** 1 Department of Mechanical Engineering, Stanford University, Stanford, CA USA; 2 Department of Aeronautics and Astronautics, Stanford University, Stanford, CA USA; 3 Department of Bioengineering, Stanford University, Stanford, CA USA

**Keywords:** Energy expenditure, Estimation, Machine learning, Gait, Ground reaction forces, Electromyography

## Abstract

**Background:**

Estimating energy expenditure with indirect calorimetry requires expensive equipment and several minutes of data collection for each condition of interest. While several methods estimate energy expenditure using correlation to data from wearable sensors, such as heart rate monitors or accelerometers, their accuracy has not been evaluated for activity conditions or subjects not included in the correlation process. The goal of our study was to develop data-driven models to estimate energy expenditure at intervals of approximately one second and demonstrate their ability to predict energetic cost for new conditions and subjects. Model inputs were muscle activity and vertical ground reaction forces, which are measurable by wearable electromyography electrodes and pressure sensing insoles.

**Methods:**

We developed models that estimated energy expenditure while walking (1) with ankle exoskeleton assistance and (2) while carrying various loads and walking on inclines. Estimates were made each gait cycle or four second interval. We evaluated the performance of the models for three use cases. The first estimated energy expenditure (in Watts) during walking conditions for subjects with some subject specific training data available. The second estimated all conditions in the dataset for a new subject not included in the training data. The third estimated new conditions for a new subject.

**Results:**

The mean absolute percent errors in estimated energy expenditure during assisted walking conditions were 4.4%, 8.0%, and 8.1% for the three use cases, respectively. The average errors in energy expenditure estimation during inclined and loaded walking conditions were 6.1%, 9.7%, and 11.7% for the three use cases. For models not using subject-specific data, we evaluated the ability to order the magnitude of energy expenditure across conditions. The average percentage of correctly ordered conditions was 63% for assisted walking and 87% for incline and loaded walking.

**Conclusions:**

We have determined the accuracy of estimating energy expenditure with data-driven models that rely on ground reaction forces and muscle activity for three use cases. For experimental use cases where the accuracy of a data-driven model is sufficient and similar training data is available, standard indirect calorimetry could be replaced. The models, code, and datasets are provided for reproduction and extension of our results.

## Background

The U.S. has an estimated 20 million people with ambulatory disabilities due to age, injury, disease, or congenital conditions [1]. These disabilities often result in less efficient gait patterns. Energy expenditure, or metabolic cost, is an important metric for understanding the level of effort required during motion [2, 3]. Gait retraining and assistive devices can reduce this effort and improve the ability of individuals with disabilities to participate in activities of daily living, but personalizing assistance requires accurate energy expenditure estimates. For example, optimizing gait parameters and device assistance with “human-in-the-loop" methods has significantly reduced energy expenditure for steady state walking [4–7]. These methods rely on indirect calorimetry to estimate energy expenditure, which is expensive and resource intensive [8]. Breath-by-breath measurements are noisy and not well correlated to instantaneous energy demands due to the complicated biological processes of mitochondrial dynamics in replenishing energy used by muscles [9–11]. Estimating energy expenditure with breath-by-breath measurements for any new condition (e.g., a new assistance profile) requires several minutes to achieve a steady state estimate.

Methods for energy expenditure estimation span statistical and data-driven approaches as well as techniques that model the underlying mechanics and biological processes. Initial statistical methods for estimating energy expenditure fit linear models to indirect calorimetry measurements of subjects moving at different speeds, inclines, or with additional loads [12–14]. Models based on walking mechanics accounted for subject specific information and gave accurate estimations for a narrow range of conditions [15–17]. Biomechanical simulations offer promise for energy expenditure estimation, but require detailed information such as joint kinematics, accurate musculoskeletal geometry, and other properties of the subject’s musculoskeletal system [18–21].

Researchers have also built data-driven models using wearable sensors or data that could be collected with wearable sensors, with the aim of portable energy expenditure estimation. Many different sensors have been employed, including accelerometers [22–24], inertial measurement units, [25, 26], heart rate monitors [27–29], immobile electromyography (EMG) systems [30–32], and various combinations [33–35]. With the exception of [36], these studies used linear regression and hand-designed features to estimate energy expenditure. Many of the models required minutes of data collection before performing estimation, requiring similar or more time than indirect calorimetry. While fitting informs the degree to which features correlate to energy expenditure, it does not evaluate the accuracy of the estimated energy expenditure for activity conditions or subjects not included in the correlation process. Some studies report accuracy with percent errors of roughly 30% [26] or root mean squared errors of approximately 1.2 $\frac {\mathrm {W}}{\text {kg}}$ [35] for walking conditions. Benchmarking performance for energy expenditure estimation would enable clinicians and researchers to select models that meet their required level of accuracy for specific rehabilitation or research tasks.

The goal of this project was to develop data-driven linear regression and neural network models to estimate energy expenditure using short time intervals of data and evaluate their accuracy in use cases where varying subject- and condition-specific data is available. We sought to evaluate both mean absolute percent error and the ability of the models to order the magnitude of energy expenditure across conditions. The input features to our models were electromyography and ground reaction force data. The models were validated with two datasets: (1) steady state walking with an ankle exoskeleton and (2) unassisted walking with a variety of loads and inclines. Although the datasets we used included only lab-based data, we sought to define an upper-bound on the performance of wearable-based estimation methods.

## Methods

### Datasets

In the first dataset, subjects walked with a unilateral ankle exoskeleton that provided a variety of ankle assistance profiles, referenced in this paper as assisted walking [37]. Eight subjects were tested (7 men and 1 woman; age = 25.1 ±5.1 yr; body mass = 77.5 ±5.6 kg; leg length = 0.89 ±0.03 m). Two subjects were rejected as their metabolic rate was more than two standard deviations from the mean of the subjects for many of the conditions. The data for the rejected subjects were not available and not included in this work. The subjects walked on an instrumented split-belt treadmill (Bertec, Columbus, OH) at 1.25 m ·*s*^−1^ for 8 minutes with the exoskeleton on one leg. Ground reaction force, metabolic, and EMG data were collected. The EMG system (Trigno Wireless System; Delsys, Boston, MA) targeted the medial and lateral aspects of the soleus, medial and lateral gastrocnemius, tibialis anterior, vastus medialis, biceps femoris, and rectus femoris on both legs. Metabolic metrics were recorded with wireless metabolics equipment (Oxycon Mobile; CareFusion, San Diego, CA). The exoskeleton applied 9 different assistance strategies, with varying amounts of work and torque. The measured energy expenditure values across these assistance strategies had a minimum of 269 W and maximum of 421 W, with an average of 343 W. The ground reaction forces and EMG signals were recorded at 2000 Hz, and all signals were recorded for the last 3 minutes of each condition once steady state was reached.

The second dataset investigated changes in energy expenditure when walking under loaded and incline conditions, referenced in this paper as inclined loaded walking [31, 38]. We used data from all subjects (9 men and 4 women; age = 33.7 ±9.0 yr; body mass = 68.8 ±11.5 kg) who completed both loaded and incline studies. Subjects walked on an instrumented split-belt treadmill (model TMO8I with incline; Bertec Corporation, Columbus, OH). Ground reaction force, metabolic, and EMG data were collected. The EMG system (DE-2.1; DelSys, Boston, MA) targeted the soleus, medial gastrocnemius, tibialis anterior, medial and lateral hamstrings, vastus medialis, vastus lateralis, and rectus femoris. Metabolic metrics were recorded (Quark b^2^; Cosmed, Italy). Each subject walked under four loading conditions where 0%, 10%, 20%, or 30% of their bodyweight was added with a weighted vest. For each weight condition, the incline was set to 0%, 5%, and 10% grades for 5 minutes each. Thus, 12 walking conditions were recorded for each subject. The minimum and maximum energy expenditure across these conditions were 183 W and 892 W, with an average of 478 W. The forces and EMG signals were recorded at 2000 Hz for the final 30 seconds of each condition and a metabolic measurement was collected continuously.

Separate data-driven models were trained on each dataset due to the different input signals. In summary, the assisted walking data had 22 time series signals consisting of 3 dimensional ground reactions forces for each foot and EMG signals from 8 muscles on both legs. The inclined loaded walking data had 14 time series signals due to 3 dimensional ground reaction forces for each foot and EMG signals corresponding to 8 muscles on one leg. These datasets were selected to test the energy expenditure estimation over a large range of energy expenditure values as well as a dataset with similar conditions that would be seen in a potential application of exoskeleton optimization.

### Data processing

The inputs to the model consisted of ground reaction forces and EMG signals. The force and EMG signals were filtered following standard biomechanics approaches. Both sets of signals were passed through a 4th order Butterworth filter. The force data were passed through a 30 Hz low pass filter to eliminate high-frequency noise. The EMG signals were filtered with a 30 to 500 Hz bandpass filter, rectified, filtered with a final 6 Hz low pass filter, and normalized by the maximum signal for each muscle during the normal walking trial.

The force and EMG time series data were formatted to make each sample of input data a fixed size. The first formatting method segmented the input signals by gait cycle, using the ground reaction forces to select data between right heel strikes. For a single gait cycle of approximately one second this results in a large and variable number of features to be fed into the estimation model. This variable length was converted to a fixed size of input data by dividing each input feature into a fixed number of bins, which were individually averaged. The number of bins was experimentally selected to be 30. Splitting data by gait cycle requires sensors to measure foot force or acceleration [39]. Another formatting method segmented data by fixed time intervals for use with sets of sensors that could not split the data by gait cycle. Every four seconds of data were taken as one input and downsampled to have an input length of 250.

The ground truth for energy expenditure was computed using indirect calorimetry. The energy expenditure was calculated in Watts by passing the recorded oxygen and carbon dioxide values from each breath into the Brockway equation [40]. The ground truth energy expenditure value for each condition was found by averaging over all breaths during the last two minutes of recorded data, once steady state motion was achieved.

### Model architectures

Linear regression and neural network models estimated energy expenditure per gait cycle and over fixed time intervals to enable estimation for activities or sensors that did not have clearly separable gait cycles. The energy expenditure estimation models used input signals formatted into a vector, *x*, of length *n* and output a single value, *y*. The length *n* is 30 times the number of input signals discretized by gait cycle. The linear regression models found the ordinary least squares solution for the weight vector, *a*, of length *n*, with a single intercept value *b* (i.e., *y*=*a*^*T*^*x*+*b*). Feature selection and regularization were not used to simplify the linear regression models.

We also built neural network models, common models for more complicated prediction tasks that rely on non-linear transformations to approximate any function. The neural networks varied in size from 3 to 4 layers and 300 to 1000 neurons per layer. We added dropout and L2 regularization to all layers to avoid overfitting to training data [41]. Rectified linear units were used as the activation functions for all neurons except the final fully connected layer. We trained the networks with a mean absolute error loss function. Percent error was computed by scaling the mean absolute error by the actual energy expenditure for each condition, giving a measure of relative accuracy. In order to compare to prior studies we also computed the root mean squared error (RMSE), normalized by average subject mass, although the model estimates had units of Watts.

In order to estimate energy expenditure for activities without a clear periodic feature, such as segmenting a gait cycle by heel strike, we considered models that use a fixed time interval of data. Fixed time interval estimates need to account for shifts in the time series data by using temporal models. A recurrent neural network was selected. The long short-term memory variant can capture long range dependencies and nonlinear dynamics [42]. The model tested had two long short-term memory layers of size 64, followed by a fully connected layer. A mean-squared error loss function was used in training, while evaluation used mean absolute error for consistency. The data were downsampled by averaging across every 32 sensor measurements to keep the length of the input data short enough for the model to perform well in recalling prior information. A four second interval of data recorded at 2000 Hz was downsampled to an input length of 250. The longest gait cycle duration from either dataset was approximately 1.5 seconds. A four second interval was selected to allow for at least two gait cycles to be represented so that multiple occurrences of the periodic signals were present in the input to the model. A longer interval was avoided to prevent gradient vanishing or exploding issues that can occur when training with long input sequences [42].

### Evaluating model performance

Three common use cases evaluated model performance: “novel condition”, “novel subject”, and “both-novel”. The novel condition use case simulated having some subject-specific training data available, as well as data from other subjects, and testing new conditions. Random conditions were removed (held out) from the training data, and treated as a test set to evaluate performance. These held out conditions were not necessarily the same conditions across all subjects. This use case is similar to research or clinical tests when the same subject repeats multiple experiments, making subject-specific data available from those prior experiments. The novel subject use case held out one entire subject to estimate all conditions for this new individual. This is often seen in clinical or research work when a new subject performs a standard set of activities that prior subjects have completed. A variant of the novel subject use case, “subject vertical force”, relied only on the vertical ground reaction forces and EMG to emulate the signals that could be recorded with wearable pressure insoles and EMG electrodes. Another novel subject variant, “raw subject”, used all signals but without any filtering other than rectifying the EMG signals. The both-novel use case held out one subject and the same conditions across all subjects. The both-novel use case represents an ideal case where a model generalizes energy expenditure estimation for a new subject completing a new task, neither of which are available in the training data. Training data for similar tasks are included.

A portion of the dataset, the validation set, was removed from the training data to tune the parameters of the neural network models. The novel condition use case held out approximately 10% of the total conditions from any subjects. The novel subject use case held out three subjects from the inclined loaded data and two subjects from the assisted data. The both-novel use case held out two complete subjects and two conditions across all subjects. The parameter values with the best performance on the validation set were selected and the validation set was placed back into the training set, due to the small number of subjects available.

The averaged model performance was measured using cross-validation. The entire dataset was divided into a number of sections equal to the number of subjects. Each section was iteratively treated as the test set, with the remaining sections combined to become the training set. The estimation accuracy was averaged across all test sets. Each test set for the novel condition use case consisted of roughly 10% of the total conditions from any subjects. The novel subject use case treated each subject as an individual test set. The both-novel use case treated each subject and two random conditions removed from all subjects as one test set; only these two conditions removed from the training data were estimated for the subject in the test set.

For a task such as exoskeleton optimization, the energy expenditure model is required to determine which assistance conditions perform the best in order to direct the optimization process towards the best performing assistance parameters. Thus, we evaluated how the models performed in ordering the magnitude of energy expenditure. Confusion matrices were used to visualize the difference in ordering the magnitude of energy expenditure across conditions for the model estimates and experimental measurements. The ground truth energy expenditure value of all conditions was ordered by increasing value along the vertical axis. The horizontal axis displayed the ordering estimated by the neural network models. The value of each grid square was normalized by the total number of subjects, thus a value of 1 corresponds to a perfect match between the measured and estimated ordering for that condition.

When ordering conditions that are close in energy expenditure value, as occurred frequently in the assisted dataset, switching their order does not constitute a meaningful error. In this case, it is preferale to consider the conditions to be equal for ordering purposes. A pairwise comparison between all estimated energy expenditure values for a single subject evaluated the ordering accuracy. The estimate for the first condition in each pair was determined to be one of three outcomes: greater than, within, or less than a threshold from the estimate of the second condition. This was repeated for the actual measured energy expenditure values. A percent ordering was determined from how many of the estimated outcomes matched the actual outcomes. The threshold level was chosen to be 4.2%, which is the mean error of estimation techniques that have been used in human-in-the-loop optimization of exoskeleton assistance [4, 7] using two minutes rather than the standard six minutes of indirect calorimetry data.

## Results

**Fig. 1:**
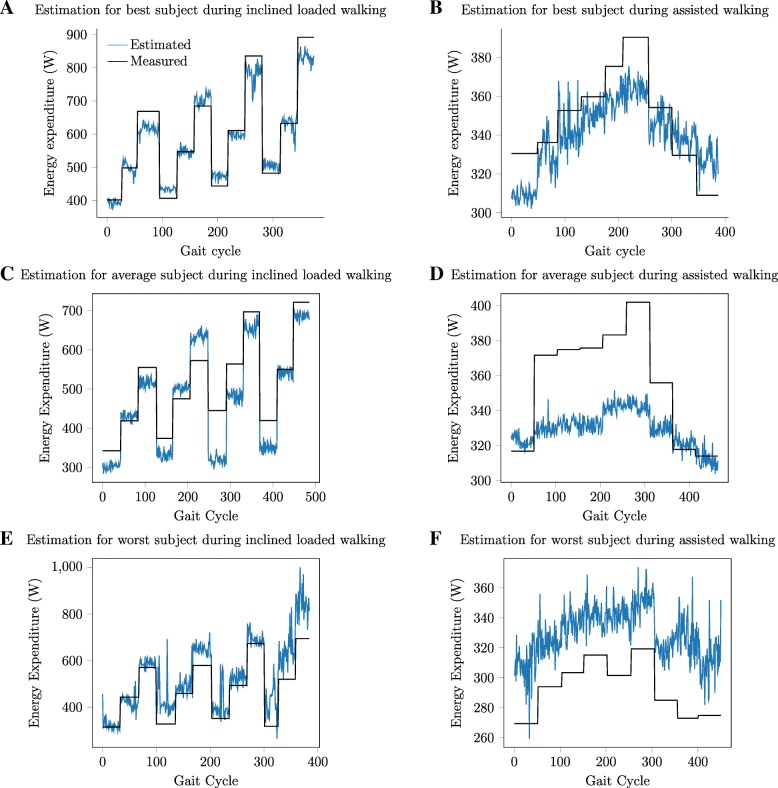
**a** Estimation for best subject during inclined loaded walking. **b** Estimation for best subject during assisted walking. **c** Estimation for average subject during inclined loaded walking. **d** Estimation for average subject during assisted walking. **e** Estimation for worst subject during inclined loaded walking. **f** Estimation for worst subject during assisted walking. A visual comparison of neural network model estimates and measured energy expenditure values for the best subject with lowest error, average subject with representative error, and worst subject with highest error when estimating all conditions in the dataset for a new subject

Data-driven models estimated energy expenditure during assisted walking and inclined loaded walking. Visualizations of the estimates for subjects with the lowest, average, and highest errors help illustrate the performance of the models (Fig. 1). These estimates occurred per gait cycle and estimated all conditions for a new subject not included in the training data.

The neural network models that estimated energy expenditure per gait cycle for a novel assisted walking condition performed best with an error rate of 4.4% (0.24 RMSE). The error rate increased to approximately 8% (0.4 RMSE) when the subject or both the subject and condition were novel (Table 1). The linear regression models performed similarly to the neural network models. The average errors for the neural network model estimates during inclined loaded walking were worse than assisted walking in all use cases (Table 2). The linear regression models performed only slightly worse, with an increase in the percent error between 0.6% and 2.4% compared to the neural network models. The R^2^ values for the linear regression models in all use cases during assisted and inclined loaded walking were greater than or equal to 0.96 when fitting training data. The recurrent neural network that used a fixed time interval of input data performed similarly to the per gait cycle model, with an average error of 8.9% and 0.4 RMSE during assisted walking. The recurrent neural network model was only used on the assisted dataset due to the significant computation required to train the models.

**Table 1:** Comparison of linear regression and neural network energy expenditure estimates made per gait cycle for different use cases during assisted walking

Model	Metric	Novel Condition^a^	Novel Subject^b^	Both-Novel^c^
Linear Regression	RMSE^d^$\left (\frac {\mathrm {W}}{\text {kg}}\right)$	0.18	0.43	0.41
	Error	4.1%	8.4%	8.2%
Neural Network	RMSE $\left (\frac {\mathrm {W}}{\text {kg}}\right)$	0.24	0.40	0.43
	Error	4.4%	8.0%	8.1%

^a^The novel condition use case randomly selected 10% of the conditions from any subjects as a test set, this was repeated as many times as there were subjects, with performance averaged across test sets

^b^The novel subject use case removed one subject at a time from the training set to be the test set, averaging the performance across all subjects

^c^The both-novel use case removed one subject at a time as well as two random conditions across all subjects from the training set. These removed conditions were estimated for the test set subject, with results averaged across all test sets

^d^RMSE is the root mean squared error normalized by the average subject mass

**Table 2:** Comparison of linear regression and neural network energy expenditure estimates made per gait cycle for different use cases during inclined loaded walking

Model	Metric	Novel Condition	Novel Subject	Both-Novel	Subject Vertical Force^a^	Raw Subject^b^
Linear Regression	RMSE^c^$\left (\frac {\mathrm {W}}{\text {kg}}\right)$	0.62	0.94	0.95	0.98	1.39
	Error	6.7%	12.1%	13.7%	12.3%	16.5%
Neural Network	RMSE $\left (\frac {\mathrm {W}}{\text {kg}}\right)$	0.56	0.83	0.78	0.86	0.88
	Error	6.1%	9.7%	11.7%	10.0%	11.2%

^a^The subject vertical force use case was the novel subject use case with inputs restricted to vertical ground reaction forces and EMG signals

^b^The raw subject use case was the novel subject use case without any data preprocessing other than rectifying the EMG signals

^c^RMSE is the root mean squared error normalized by the average subject mass

A recurrent neural network using a fixed time interval of input data had an average error of 8.9% and MAE of 0.52 $\frac {\mathrm {W}}{\text {kg}}$ when estimating energy expenditure during all assisted walking conditions for a new subject. The recurrent neural network resulted in a 19.5% increase in MAE compared to the neural network estimating all conditions for a new subject per gait cycle.

**Fig. 2:**
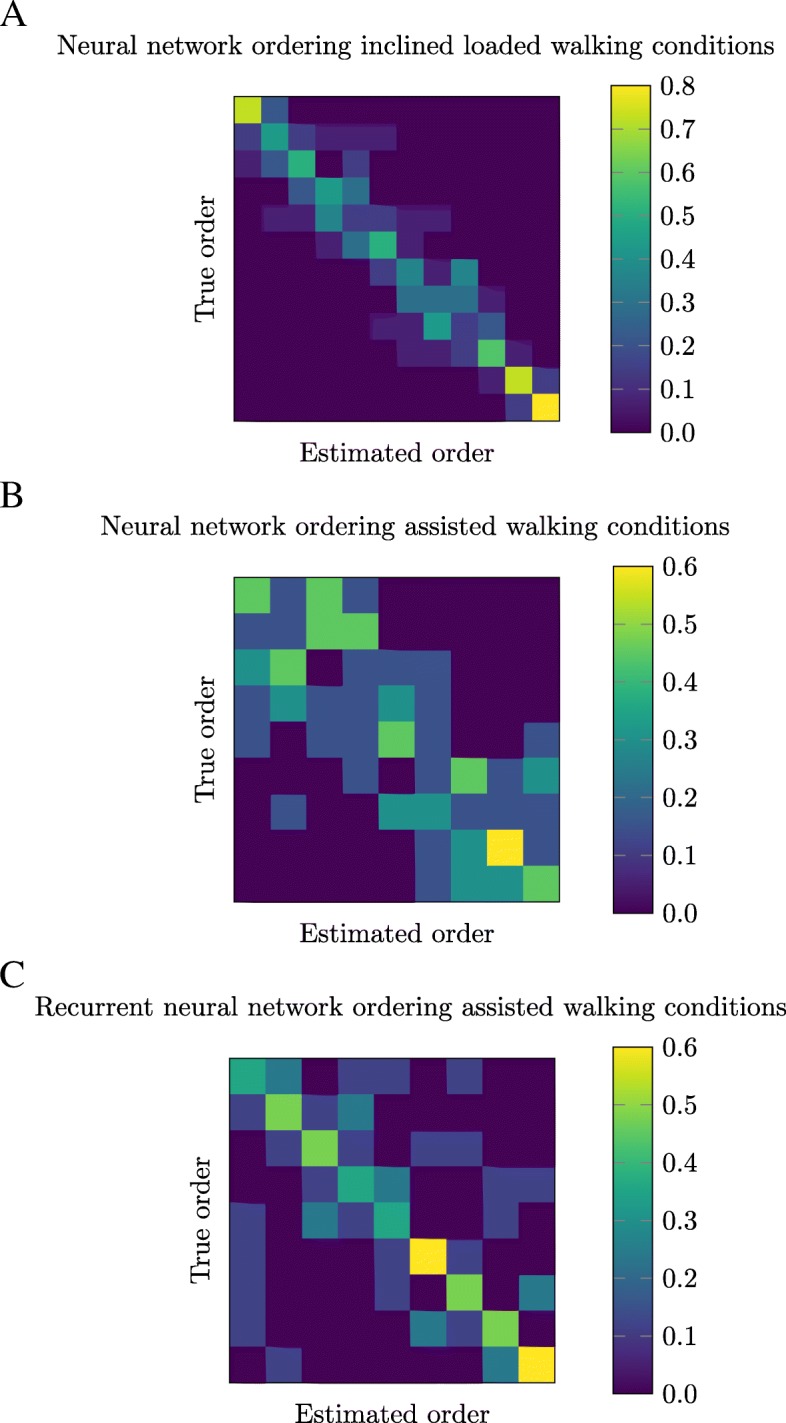
**a** Neural network ordering inclined loaded walking conditions. **b** Neural network ordering assisted walking conditions. **c** Recurrent neural network ordering assisted walking conditions. Visualized differences between the ordering of true, or measured, energy expenditure and the estimations across all conditions in the dataset for new subjects. The value in each grid square represents the number of estimated conditions ordered to match the corresponding true energy expenditure value. Perfect ordering results in a diagonal trend

The neural network models ordered the energy expenditure across all inclined and loaded walking conditions with a clear diagonal trend (Fig. 2a). The neural network ordering during assisted walking was less accurate, with additional errors causing a noisier diagonal trend (Fig. 2b). The recurrent neural network improved the clarity of the diagonal trend during assisted walking, but increased the spread of the outliers (Fig. 2c). Using pairwise comparison to evaluate the ordering, the neural network models achieved an average percentage of correctly ordered conditions of 87% and 61% for inclined loaded and assisted walking. The recurrent neural network slightly improved the performance averaging 63% correctly ordering conditions for assisted walking.

Restricting the input signals to EMG and the vertical ground reaction force increased the error minimally compared to using all input signals when estimating energy expenditure for new subjects (Table 2). Using all input signals but not performing any data processing other than rectifying the EMG signals increased the error for linear regression by 36% and neural networks by 15% compared to processed inputs.

Models restricting the inputs to only ground reaction forces performed similarly to including all signals and barely outperformed models restricting the inputs to only EMG signals during assisted walking with a neural network (Table 3). Linear regression achieved similar levels of performance. For inclined loaded walking, models using only ground reaction force as inputs significantly outperformed models using only EMG inputs with roughly half the error.

**Table 3:** Average error for models with input features of either ground reaction forces or EMG signals when estimating energy expenditure during all conditions in the dataset for a new subject

Dataset	Model	Forces^a^	EMG^b^
Assisted	Linear Regression	8.7%	9.4%
	Neural Network	8.1%	9.2%
Incline-load	Linear Regression	12.5%	31.6%
	Neural Network	11.5%	23.9%

^a^The model’s input features were restricted to only include the ground reaction forces, excluding the EMG signals

^b^The model’s input features were restricted to only include the EMG signals, excluding the ground reaction forces

## Discussion

The purpose of this project was to validate a method of estimating energy expenditure per gait cycle or short interval of data using input signals possible to measure with wearable sensors. Two representative datasets using immobile versions of these features were selected to have a large range of energy expenditure values across conditions (inclined loaded dataset) and similar conditions to those encountered during exoskeleton optimization (assisted dataset). Using immobile sensors rather than wearable versions offers an upper bound on the model performance before requiring specific wearable sensors to be selected and enables the use of such models in clinics or labs. These models predict with higher accuracy than previous models and perform well even with unfiltered inputs or using only ground reaction forces as inputs. The models are able to capture the relative changes in energy expenditure following the trends of the measured energy expenditure across the range of conditions, even for subjects with average or the worst performance (Fig. 1). The ability to capture relative changes between conditions enables the models to order the magnitude of energy expenditure across multiple conditions.

When estimating energy expenditure for a novel condition, the RMSE and percent error were approximately half that of models estimating for a novel subject (Tables 1 and 2). Estimating energy expenditure for novel subjects and conditions performed similarly to the models for novel subjects. This indicates the models captured some relationship between the inputs and energy expenditure in order to account for the new conditions of a similar type without a significant increase in error, rather than just fitting a specific set of conditions found in the training data. The worse performance across all inclined loaded walking models was likely due to the larger range of energy expenditure values across conditions than during assisted walking (Table 2).

The additional trainable weights in neural networks marginally improved performance over linear regression. the neural networks used here had additional trainable weights and the ability to capture nonlinear relationships, which could offer improved performance when more data is available, or when there is more heterogeneity in the conditions presented. For example, the similar performance of the neural networks trained on raw or processed inputs shows promise for handling noisier wearable sensor data with minimal preprocessing. The high R^2^ values from fitting linear regression training data indicated that using EMG and ground reaction forces as inputs with the binning structure was informative for estimating energy expenditure.

Several of the results of our testing indicate that our approach could be suitable for extension to new types of sensors, including purely wearable sensors. The minimal increase in error when restricting inputs to the vertical ground reaction force and EMG signals indicated wearable sensors measuring normal force, such as pressure sensing insoles, could be capable of collecting the most important force information (Table 2). Wearable pressure sensor insoles were found to have an RMSE between 6.6% and 17.7% from ground reaction forces during activities such as walking, standing, or lifting weights [43]. The recurrent neural network with fixed time intervals of input data performed slightly worse than the per gait cycle estimation, but could enable flexibility for use during activities without a clear periodic structure.

Capturing the general trends in energy expenditure across conditions is important for distinguishing the order of effort among conditions for a subject. The large range of energy expenditure values across inclined loaded conditions could have made ordering simpler than the assisted conditions. The confusion matrix for the inclined loaded data had a clear diagonal trend with any errors occurring near the correct ordering (Fig. 2a). The confusion matrix for the assisted conditions show a less defined diagonal trend (Fig. 2b). The recurrent neural network model ordering during assisted walking improved the clarity of the diagonal trend, but with outliers further from the correct conditions (Fig. 2c).

Understanding the computation the neural networks performs is challenging, but comparing the performance of models with different input types provides some insight. Models with the input features restricted to either ground reaction forces or EMG signals had similar weights for both sets of input features during assisted walking (Table 3). Inclined loaded walking models relied more on the ground reaction forces, as these forces likely encapsulated information such as subject weight, incline, and amount of added load. Thus, the importance of certain features is dependent on the walking conditions. A wider range of features could enable more robust performance when generalizing to new conditions.

Prior work evaluated the accuracy of energy expenditure estimation of commercial wrist-worn devices that measured heart rate and accelerations at the wrist [26]. For walking and running activities, the average relative error for these devices was approximately 31%. The models required one minute intervals of data to perform estimation. This estimation task is most similar to our use case estimating new subjects and new conditions during inclined and loaded walking, which achieved 13.7% and 11.7% errors for the linear regression and neural network models. Our estimates occur at approximately one second intervals. The improved performance relative to the wrist-worn sensors is likely due to the additional information gathered by placing sensors on the lower limbs.

Another energy expenditure study used a combination of wearable and immobile sensors including accelerometers, EMG, skin temperature, heart rate, minute ventilation, and breathing frequency to estimate energy expenditure using linear regression [35]. A total of 20 conditions were tested for activities including resting, walking, running, and cycling. These conditions had an energy expenditure range of 10.0 $\frac {\mathrm {W}}{\text {kg}}$, similar to the 10.3 $\frac {\mathrm {W}}{\text {kg}}$ range in the inclined loaded dataset. When estimating new conditions using all sensors, their RMSE was 1.28 $\frac {\mathrm {W}}{\text {kg}}$. The RMSE when estimating new conditions for the inclined loaded dataset with a linear regression model was 0.62 $\frac {\mathrm {W}}{\text {kg}}$, with similar R^2^ values for both models. The smaller RMSE in this study indicates the gait structure and vertical ground reaction force offer additional information that can improve accuracy and show similar correlation to the wearable sensors in the prior study.

### Limitations

These data-driven models could be improved by fine tuning the features and including more wearable sensor measurements. The linear regression features consisted of input signals individually averaged into a fixed number of bins for each gait cycle. Using feature selection or hand designing additional features could improve the performance of linear regression.

The three directions of ground reaction forces used in some of the trained models cannot be measured with wearable sensors, but prior work used force sensing insoles to estimate the three directions accurately [44]. Using wearable sensors could add noise which would likely reduce performance. In order to investigate the efficacy of this method for performance during activities outside a lab setting, a study using completely wearable sensors to estimate energy expenditure over many different conditions and subjects would be necessary.

In order to select hyperparameters for the neural network models, the validation set is typically completely separated from the training and testing data. As an example, in the use case where a new subject and condition were estimated this would require the removal of approximately 16% of the inclined loaded dataset and 25% of the assisted dataset. Only two conditions for two subjects were used in this validation process, approximately 2.6% of the inclined loaded dataset and 5.6% of the assisted dataset. Due to the small size of the datasets, we included the validation set for cross-validation. By averaging the results from a number of folds equal to the number of subjects we expected the potential impact of incorporating prior knowledge from less than 6% of the datasets would be minimal compared to the change in performance by removing 16 to 25% of the available data.

The similarity between the conditions in the training set and test set impacts performance. When applying these models to other datasets, the same level of performance is expected if the new dataset has a similar size. A truly generalizable model for energy expenditure estimation would require significantly more data across a wider range of conditions and subjects. Hand designed experiments showed that estimating conditions with energy expenditure levels on the extremes of the dataset were the most difficult. For example, holding out the two conditions where the exoskeleton applied the most work resulted in roughly three times the error compared to holding out random conditions with a linear regression model for the both-novel use case. To use these models in practice, the range of conditions to be tested should be similar to the conditions in the training data. In general, these models do not estimate the absolute energy expenditure as accurately as indirect calorimetry. For practical use, the trade-off between the speed and accuracy of the estimates will need to be considered.

## Conclusions

This work benchmarks the performance of models used to rapidly estimate energy expenditure for use cases common in clinical and rehabilitation settings. If the performance of a model described here meets the requirement of a particular study and the biomechanical conditions to be tested are similar to those in the data sets used for training, researchers could use these models rather than indirect calorimetry. Researchers could also train their own models to estimate for new conditions. The similar accuracy when estimating energy expenditure for conditions present in the dataset for a new subject and new conditions for a new subject suggests that the models learned a relationship between the input features and the energy expenditure, rather than just fitting conditions with similar training data. The models were also able to order the energy expenditure across conditions which could enable selection of optimal assistance conditions. Restricting input features to signals possible to measure with wearable sensors allows for scalable deployment of the models, but requires validation with completely wearable sensors. These models take steps towards generalizable energy expenditure estimation which could be used in interventions that improve rehabilitation and mobility beyond a lab setting.

## Abbreviations

EMG: Electromyography
RMSE: Root mean squared error
